# Single/low-copy integration of transgenes in *Caenorhabditis elegans *using an ultraviolet trimethylpsoralen method

**DOI:** 10.1186/1472-6750-12-1

**Published:** 2012-01-05

**Authors:** Eriko Kage-Nakadai, Hiroyuki Kobuna, Osamu Funatsu, Muneyoshi Otori, Keiko Gengyo-Ando, Sawako Yoshina, Sayaka Hori, Shohei Mitani

**Affiliations:** 1 Department of Physiology, Tokyo Women's Medical University School of Medicine, Tokyo, Japan; 2 Core Research for Evolutional Science and Technology (CREST), Japan Science and Technology Agency (JST), Saitama, Japan; 3 Saitama University Brain Science Institute, Saitama, Japan

## Abstract

**Background:**

Transgenic strains of *Caenorhabditis elegans *are typically generated by injecting DNA into the germline to form multi-copy extrachromosomal arrays. These transgenes are semi-stable and their expression is silenced in the germline. Mos1 transposon or microparticle bombardment methods have been developed to create single- or low-copy chromosomal integrated lines. Here we report an alternative method using ultraviolet trimethylpsoralen (UV/TMP) to generate single/low-copy gene integrations.

**Results:**

We successfully integrated low-copy transgenes from extrachromosomal arrays using positive selection based on temperature sensitivity with a *vps-45 *rescue fragment and negative selection based on benzimidazole sensitivity with a *ben-1 *rescue fragment. We confirmed that the integrants express transgenes in the germline. Quantitative PCR revealed that strains generated by this method contain single- or low-copy transgenes. Moreover, positive selection marker genes flanked by LoxP sites were excised by Cre recombinase mRNA microinjection, demonstrating Cre-mediated chromosomal excision for the first time in *C. elegans*.

**Conclusion:**

Our UV/TMP integration method, based on familiar extrachromosomal transgenics, provides a useful approach for generating single/low-copy gene integrations.

## Background

The development of methods to introduce exogenous DNA into animals has allowed for diverse genetic manipulations in many organisms. In *Caenorhabditis elegans*, transgenic strains are typically generated by injecting DNA into the syncytial germ cells of the hermaphrodite gonad to form multi-copy extrachromosomal arrays [1]. These transgenes are semi-stable; transgenic animals are mosaic in that some cells lose the extrachromosomal array, and transmission of arrays to the next generation is partial [2]. Extrachromosomal arrays contain hundreds of copies of the injected DNA [1], leading to silencing of the transgene expression in the germline [3]. Although extrachromosomal arrays can be integrated into the chromosomes by gamma-ray irradiation or ultraviolet (UV) [4,5], integrated arrays still contain a high copy-number of transgenes that seldom escape gene silencing.

Methods using microparticle bombardment were developed to create low-copy chromosomal integrated lines [6]. The biolistic technique allows for direct integration of small amounts of exogenous DNA into the chromosomes, avoiding the formation of extrachromosomal arrays. Not every bombardment, however, produces integrant animals because of the low frequency of events, although large number of animals can be bombarded at once (~10^4^/bombardment) [6]. More recently, techniques using Mos1 transposons were developed and are frequently used to generate single-copy gene insertions [7]. This technique, called Mos1-mediated single-copy insertion (MosSCI), is based on homologous recombination: A double-stranded break in the chromosome mediated by Mos1 excision is repaired with an exogenously supplied template carrying the gene of interest and homology arms, generating the designed single copy insertion [7]. MosSCI methods, in which a recipient strain carrying a Mos1 element is microinjected with a targeting vector and a Mos1 transposase expression vector, exhibit a high frequency of insertion, and injection of 20 worms is enough to obtain integrant animals [7]. In the MosSCI method, large sized targeting vectors that contain positive selection marker, 5'- and 3'- homology arms, and gene of interest must be constructed for each insertion, although a Gateway-compatible tool kit for MosSCI has been developed [8].

In reverse genetic studies, ultraviolet trimethylpsoralen (UV/TMP), which induces a small deletion in the chromosomes, has been widely used to generate deletion mutants [9-11]. In addition to deletions, insertions of unexpected DNA fragments are often observed, suggesting that non-homologous DNA is used as a template in end-joining repair mechanisms. UV irradiation (wavelength 365 nm) and TMP treatment has a higher mutation frequency and less rearrangement of chromosomes, such as inversion and translocation, compared to UV irradiation (wavelength 254 nm), which is used for insertion of extrachromosomal arrays [5,11,12].

In the present study, we developed a technique using UV/TMP that produces single- or low- copy gene integrations from extrachromosomal arrays. In this method, we used a positive selection marker flanked by LoxP sites, allowing the marker to be excised by the Cre recombinase.

## Results and discussion

### Transgene integration using UV/TMP methods

Our methods were based on random integrations of transgenes into the chromosomes from multi-copy extrachromosomal arrays. We adopted a new positive-negative selection strategy as follows: Positive selection was based on rescue of the *vps-45 *mutant phenotype. *vps-45 *mutants are unable to grow and reproduce normally at 20°C [13]. As a result, only *vps-45 *mutants carrying the positive selection marker, the *vps-45 *mini gene, grow and reproduce, allowing for easy identification of the transformants. To discriminate single- or low-copy integrations from extrachromosomal arrays and multi-copy integrations, we used *ben-1 *as a negative selection marker. Mutants of *ben-1*, which encodes a ß-tubulin of *C. elegans*, are resistant to an anti-tubulin drug benzimidazole: mutants grow paradoxically more quickly than wild-type animals, which are severely unhealthy, dumpy, and uncoordinated in movement on benzimidazole-containing selection media [14]. Thus, *ben-1 *mutants not carrying the negative selection marker, the *ben-1 *gene, predominantly grow and reproduce on the selection media, enabling differentiation of low copy integrants from Ex arrays and multi-copy integrants that are highly likely to have the *ben-1 *gene (Additional file 1, Figure S1).

**Figure 1:**
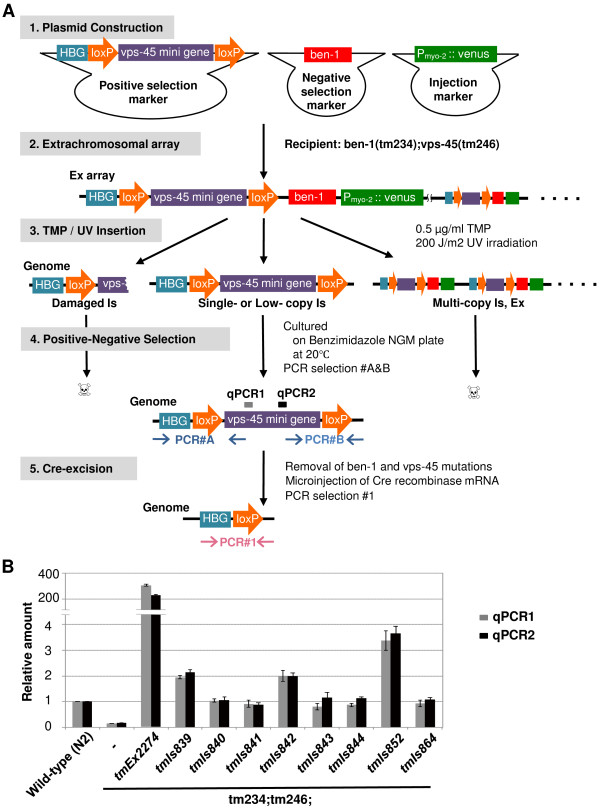
**Schematic overview of UV/TMP low-copy integration**. (A) Positive selection marker (*vps-45 *mini gene), negative selection marker (*ben-1 *genome) and injection marker (*P*_*myo-2*_*::venus*) were co-injected into the recipient strain (*tm234(ben-1);tm246(vps-45)*) to generate transgenic strains carrying Ex arrays. Ex strains were treated by UV/TMP to obtain insertion (Is) strains. Is strains were cultured at 20°C on benzimidazole-containing NGM plates. Under these conditions, single- or low- copy integrated strains survived but multi-copy integrants or Ex array-carrying animals did not survive due to multi-copy transgenes containing the *ben-1 *gene. Strains were further tested by PCR#A and PCR#B to identify the intact floxed *vps-45 *mini gene. PCR-selected lines were backcrossed with N2 wild-type to remove *vps-45 *and *ben-1 *mutations, followed by microinjection of Cre recombinase mRNA to excise the *vps-45 *mini gene. Cre-LoxP excision was detected by PCR#1. qPCR1 and qPCR2 indicate regions amplified by quantitative PCR described below. (B) Relative amount of the *vps-45 *gene determined by quantitative PCR with primers that amplify the 5' region in the *vps-45 *mini gene (gray bars: qPCR1) and the middle region (black bars: qPCR2). The amount of the *vps-45 *gene (normalized to the *act-2 *gene) is presented as a ratio to the N2 control. Error bars represent SE of three independent experiments.

We prepared a positive selection marker and a negative selection marker, as shown in Figure 1. The positive selection marker included a floxed *vps-45 *mini gene and partial human *ß-globin *(HBG) tag sequence to detect the insertion. The negative selection marker contained the *ben-1 *genomic sequence. Extrachromosomal (Ex) arrays were generated by microinjection of the positive and negative selection plasmids with an injection marker into *tm234(ben-1);tm246(vps-45)*. To insert the extrachromosomal transgenes into the chromosomes, animals carrying Ex arrays were treated with UV/TMP to induce a small deletion in the chromosomes, leading to insertion during gap repair. UV/TMP-treated animals were cultured on benzimidazole-containing media at 20°C for positive-negative selection. To identify integrants that retained an intact insertion sequence, we conducted PCR#A and PCR#B, which amplify the 5' and 3' regions, respectively, of HBG-LoxP-vps-45-LoxP (Figure 1A).

### Frequency of integrations

We used *tm234;tm246;tmEx2274 *as a parent Ex strain and performed three independent UV/TMP integration methods. Approximately 16,000, 10,400, and 10,400 P0 animals from the parent Ex strain were treated with UV/TMP. We then collected 176,000, 50,400, and 146,000 F1 animals, and cultured them under the selection conditions. As a result, 53, 32, and 56 stable transformants were obtained. Isolated transformants were further tested by PCR#A and PCR#B, resulting in 22, 19, and 22 integrated strains (Table 1, HBG#1-3). The mean integration frequency (integrants/P0 animals) was 0.17. The UV/TMP method produced a much higher frequency than the bombardment protocol (Additional file 2, Table S1) [6].

**Table 1:** Frequency of integrations using UV/TMP methods

**Exp**.	P0	F1	Transformants	PCR selection	Frequency
					(Is lines/P0 animals)
HBG#1	16,000	176,000	53	22	0.13
HBG#2	10,400	50,400	32	19	0.18
HBG#3	10,400	146,000	56	22	0.21
HSP	16,000	110,000	12	4	0.025

### Germline expression of transgenes

**Figure 2:**
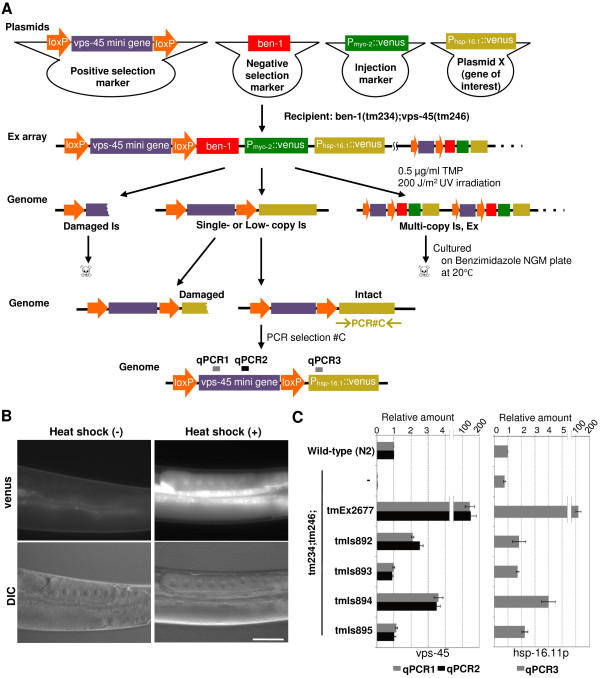
**Germline expression of integrated transgenes**. (A) *P*_*hsp-16.1*_*::venus *was co-injected with a positive-selection marker, a negative-selection marker and an injection marker. Ex array-carrying transgenic animals were treated by UV/TMP. F1 animals were cultured under the condition of positive-negative selection. PCR selection with primers to detect the intact *P*_*hsp-16.1*_*::venus *transgene (PCR#C), determined the integrant lines. qPCR3 indicates the amplified region by quantitative PCR described below. (B) Venus protein was expressed in germ cells by heat shock, suggesting that low-copy transgenes were successfully integrated. *tmIs894 *adult hermaphrodites are presented. Scale bar = 50 μm. (C) Relative amount of the *vps-45 *gene determined by quantitative PCR with primers that amplified the 5' region in the *vps-45 *mini gene (gray bars: qPCR1) and the middle region (black bars: qPCR2; left graph), and relative amounts of the *hsp-1.2 *promoter region (gray bars: qPCR3; right graph). The amount (normalized to the *act-2 *gene) is presented as a ratio to the N2 control. Error bars represent SE of three independent experiments.

To validate the UV/TMP methods, we used a heat shock-inducible *Venus *plasmid (*P*_*hsp-16.1*_*::venus*) as a tester plasmid. We co-injected *P*_*hsp-16.1*_*::venus *with selection markers and an injection marker, generating a parent Ex strain *tm234;tm246;tmEx2677*. Approximately 16,000 P0 animals were treated by UV/TMP and cultured for positive-negative selection as described above, resulting in 12 transformants. To identify integrated strains retaining the intact *P*_*hsp-16.1*_*::venus *sequence, we performed PCR#C, which amplifies the whole region of *P*_*hsp-16.1*_*::venus *(Figure 2A). As a result, four integrant strains were isolated (Table 1, HSP). The relative low frequency (0.025) compared to that of HBG#1-3 (0.17 on average) was probably due to the length of the transgene to be inserted. Next, we tested whether transgenes can be expressed in germ cells, because high-copy transgenes are usually silenced in the germline. Four integrants were heat-shocked at 32°C for 1 h, followed by culture at 20°C for 3 h. As a result, Venus protein expression in the germline was observed in all four integrant strains (*tmIs893 *was presented in Figure 2B). We also analyzed the *venus *mRNA level by quantitative RT-PCR, which showed an extensive induction (Additional file 3, Figure S2). These findings suggested that these strains contain low-copy or single-copy of transgenes.

### Copy number of integrated transgenes

To determine the copy number of insertions, we performed quantitative PCR using purified genome DNA as a template. We designed primer sets located within an exon of the *vps-45 *gene to detect both endogenous *vps-45 *and exogenous *vps-45 *mini gene insertions. Eight HBG integrant strains were tested and compared to wild-type N2, which has two copies of the *vps-45 *gene. As a result, five strains in the *tm234(ben-1);tm246(vps-45)*-background: *tmIs840, tmIs841, tmIs843, tmIs844*, and *tmIs864 *showed almost the same relative amount as N2, suggesting that these strains had two copies of the *vps-45 *gene, probably because they were homozygous for alleles carrying a single insertion site (Figure 1B). On the other hand, two strains in the *tm234(ben-1);tm246(vps-45)*-background: *tmIs839, tmIs842 *showed two times higher amounts, and one strain in the *tm234(ben-1);tm246(vps-45)*-background: *tmIs852 *showed three or four times higher amounts compared to N2 (Figure 1B), suggesting that these strains contain a low-copy, but not a single-copy number, of transgenes. Examination of *tm234;tm246;tmEx2274*, the parent strain, revealed that the parent Ex strain contained hundreds of copies of transgenes (Figure 1B), consistent with previous studies [1]. These results strongly suggest that UV/TMP methods produce single- or low-copy integrations. We also determined the copy number of the *vps-45 *gene in four HSP integrated strains, revealing the single copy insertion of the *vps-45 *mini gene in *tmIs893 *and *tmIs895 *and low-copy, but not single-copy insertion, in *tmIs892 *and *tmIs894 *(Figure 2C, left). We also examined the copy number of *P*_*hsp-16.1*_*::venus *using primers that amplify the *hsp-16.1 *promoter region, and found that *tmIs892, tmIs893, tmIs895 *contained a single-copy insertion of *P*_*hsp-16.1*_*::venus *(Figure 2C, right). These results demonstrate that the UV/TMP method is useful for low-copy insertion of genes of interest and also highly likely to produce single-copy insertion.

### Genomic-excision using Cre mRNA microinjection

**Figure 3:**
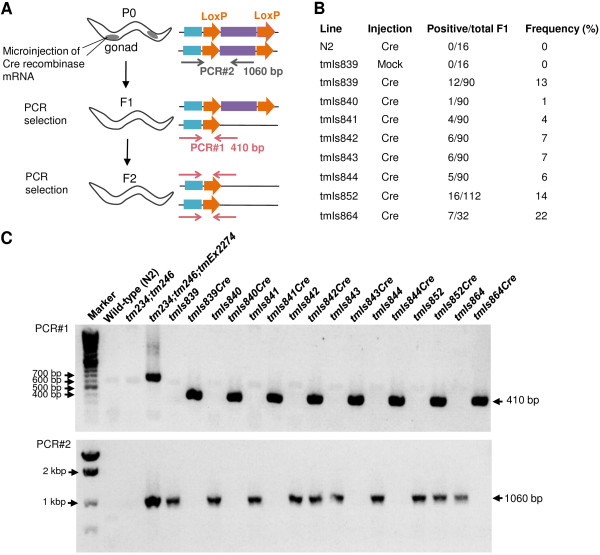
**Genomic exicision of positive selection markers using Cre recombinase mRNA**. (A) A schematic overview of Cre-mediated excision. Cre recombinase mRNA was microinjected into the gonads of P0 hermaphrodites carrying homozygous *vps-45 *mini genes flanked by LoxP sites. F1 hermaphrodites were examined by PCR#1 in which Cre-LoxP excision resulted in 410-bp products. Self-progenies (F2 hermaphrodites) were examined by PCR#1 and PCR#2 to confirm that *vps-45 *mini genes were successfully excised. (B) Frequency of excision (PCR#1 positive/total F1) by microinjection of Cre mRNA. (C) Cre-excision was detected by PCR#1(upper, 410 bp). Unexcised regions were detected by PCR#2 (lower, 1060 bp).

The eight HBG strains tested above were outcrossed with N2 to remove *ben-1 *and *vps-45 *mutations, and side mutations caused by UV/TMP treatment. These strains were further microinjected with Cre recombinase mRNA to excise the floxed *vps-45 *mini gene. F1 hermaphrodites from injected P0 animals were isolated and tested by PCR#2, in which the 410-bp band was amplified only when an excision successfully occured (Figure 3A). The results indicated that all eight strains produced F1 animals carrying a Cre/LoxP-excised region. Although the frequency varied among strains, excision occurred at 7.6% (PCR#2-positive F1 animals/P0 animals injected) on average (Figure 3B). All trials using at least two independently purified Cre mRNA produced excision in all strains tested (data not shown). Homozygotes for excised alleles were selected from F2 self-progenies of each strain, and assayed by PCR#2 as described above and PCR#3, which produced a 1060-bp band when an unexcised region was present. As a result, *tmIs839Cre, tmIs840Cre, tmIs841Cre, tmIs843Cre, tmIs844Cre*, and *tmIs864Cre *(we refer to the Cre-excised strain from original strain *tmIsxxx *as *tmIsxxxCre*) produced 410-bp bands with PCR#2 and no band with PCR#3, indicating complete excision of the LoxP sites (Figure 3C). We confirmed that Cre-untreated strains corresponding to these Cre-treated strains showed 1060-bp bands with PCR#3 (Figure 3C). On the other hand, *tmIs842Cre *and *tmIs852Cre *showed both products of PCR#2 and #3, indicating the an unexcised region remained, although at least one excision occurred. A band of 600 to 700 bp observed in *tm234;tm246;tmEx2274 *was thought to be a product of the rearranged Ex arrays. Of six strains with complete excision, five strains were predicted to have single-copy insertions based on the quantitative PCR results. One strain, *tmIs839*, was predicted to have a few copies of the insertion, suggesting that insertion sites were segregated during outcrossing resulting in isolation of a single-copy insertion strain, or that a few LoxP sites were simultaneously excised or both.

### Mapping

To determine the insertion sites and confirm chromosomal-excision (but not extrachromosomal-excision) of the LoxP sites, we performed single nucleotide polymorphism (SNP) mapping, in which linkage between the HBG-loxP sequence (produced by Cre-mediated excision and detected by PCR#2) and SNPs. We examined *tmIs840Cre, tmIs841Cre, tmIs843Cre, tmIs844Cre*, and *tmIs864Cre*, which originally contained a single-copy insertion of the transgenes and showed complete excision of the floxed *vps-45 *mini gene. Cre-excised sites were mapped to I:-12~-1, II:-18~1, III:4~12, X:8~11, and X:8~17 in *tmIs840Cre, tmIs841Cre, tmIs843Cre, tmIs844Cre*, and *tmIs864Cre*, respectively (Table 2). *tmIs839Cre*, which originally had multi-copy insertion(s) but showed a complete excision pattern, was also tested, and showed a linked chromosomal position at IV:1~14 (Table 2). These insertion sites suggest that the transgenes were randomly integrated in the chromosomes. We also mapped the insertion sites of original strains with a *tm234;tm246*-background, and confirmed compatible insertion sites (data not shown). These results indicated that chromosomal insertion of the floxed *vps-45 *mini gene was excised by Cre recombinase.

**Table 2:** Mapping of Cre-excised insertion sites

Alleles			Chromosome			
	I	II	III	IV	V	X
tmIs839Cre	U	U	U	1~14	U	U
tmIs840Cre	-12~-1	U	U	U	U	U
tmIs841Cre	U	-18~1	U	U	U	U
tmIs843Cre	U	U	4~12	U	U	U
tmIs844Cre	U	U	U	U	U	8~11
tmIs864Cre	U	U	U	U	U	8~17

U: unlinked

## Conclusion

In the present study, we developed an alternative method to promote low copy/single copy integration using UV/TMP. This method is based on the familiar extrachromosomal transgenics and does not require any special equipment, and should be very easy for most *C. elegans *researchers. Our methods exhibited higher efficiency when compared to the microparticle bombardment system, and lower efficiency when compared to MosSCI (Additional file 2, Table S1). The low-copy of integrated transgenic lines rather than single-copy lines, however, may be precious in some cases, *e.g*. when proteins of interest may not be sufficiently expressed by single-copy transgenes. Indeed, a few-copies of *P*_*hsp-16.1*_*::venus *transgene exhibited brighter fluorescence and expressed several times higher *Venus *mRNA than the single-copy transgene (data not shown and Additional file 3, Figure S2). We adopted *vps-45 *as a positive selection marker and *ben-1 *as a negative selection marker, whereas previous microparticle bombardment and MosSCI systems used other selection markers (Additional file 2, Table S1). The *vps-45 *mutants showed a distinct *ts *phenotype and *ben-1 *mutants exhibited a strong benzimidazole-resistant phenotype, both of which were fully rescued by transgenes, enabling desired integrants to be easily identified. Our selection strategy offers a new gene set for positive-negative selection in many other screens. We used a floxed positive selection marker to be excised afterwards. Previous studies showed that Cre-mediated LoxP excision successfully occurs in the extrachromosomal arrays [15,16]. In these cases, however, excision was limited in a small portion of hundreds of floxed transgenes. Our results demonstrate Cre-mediated chromosomal and complete excision in *C. elegans *for the first time. The UV/TMP integration method provides a useful approach to generating single/low-copy gene integrations.

## Methods

### Strains

*C. elegans *strains were cultured using standard techniques [17]. The wild-type strain Bristol N2 was obtained from the Caenorhabditis Genetics Center. Strains carrying the following mutations were obtained from the UV/TMP mutagenized library, as described previously [11] and identified by PCR amplification with primers spanning the deletion region of *ben-1(tm234)III *and *vps-45(tm246)X*, as described previously [11,13]. The mutants were backcrossed four times with N2. Primers used for PCR genotyping were as follows: tm234_1^st^round, 5'-ACGTGGGAATGGAACCATGT-3', 5'-TCTCCATTTCCTCTTCCTCC-3'; tm234_2^nd^round, 5'-CTCCGGACATTGTAACGGAA-3', 5'-CCCTCCATTTGAAAGAGTCC-3'; tm246, 5'-CGCAATTGGATACTACTTGT-3', 5'-TCTCCTGCTCTACTTCTGCT-3'.

### Constructs and transgenic lines

The positive selection marker plasmid (*pFX_HBG_Lw_vps-45*) was constructed by subcloning the *HBG1 *sequence (60 bp of partial human *ß-globin *sequence), wild-type *LoxP *sequence (ATAACTTCGTATAGCATACATTATACGAAGTTAT) [18], and *vps-45 *mini gene (*eft-3p::vps-45cDNA::unc-86 3'-UTR*), into pBluescriptII MCS. The whole sequence is available upon request. The negative selection marker plasmid (*pGEMT_ben-1(+)*) was constructed by TA-cloning in which the *ben-1 *genome (2414 bp of the 5' upstream region followed by the coding sequence and 785 bp of the 3'-UTR) was subcloned into the pGEMT-easy vector. To generate the *P*_*hsp-16.1*_*::venus *plasmid (*pFX_hs::venusT*), 117 bp of the upstream genomic region of the *hsp-16.1 *gene was cloned into the 5' region of the Venus expression vector in-frame [19]. To generate *tmEx2274 *transgenic animals, *pFX_HBG_Lw_vps-45, pGEMT_ben-1*(+) (80 ng/μl) were co-injected with *P*_*myo-2*_*::venus *as an injection marker (20 ng/μl) into *tm234(ben-1);tm246(vps-45)*. To generate *tmEx2677 *transgenic animals, *pFX_HBG_Lw_vps-45, pGEMT_ben-1*(+) and *pFX_hs::venusT *were co-injected at 67 ng/μl each along with *P*_*myo-2*_*::venus *as an injection marker (20 ng/ml) into *tm234(ben-1);tm246(vps-45)*.

### UV/TMP treatment and positive-negative selection

Treatment with UV and TMP was conducted as described below. TMP (Wako) was dissolved completely in acetone at a concentration of 0.3 mg/ml and diluted to 0.5 μg/ml in M9 buffer just before use. Mixtures of young adults and L4 larvae of *tm234(ben-1);tm246(vps-45)*-background Ex lines described above (cultured at 20°C) were collected from the NGM agar plates and incubated for 1 h at room temperature in the dark at 0.5 μg/ml of TMP. The animals were irradiated with 365 nm UV with a UV hand-monitor (UVP Inc.) at 200 J/cm^2^. The intensities of the UV light were calibrated with a UV luminometer (UVP Inc.) and controlled by the exposure time. UV/TMP-treated worms were plated on NGM agar dishes and allowed to lay eggs at 20°C. After 24 h, adults and larvae were washed off to remove all the animals fertilized before treatment, and incubated at 20°C for another 24 h, while F1 animals hatched. F1 animals were collected, washed three times with M9 buffer, and plated onto NGM agar plates containing 10 μg/ml of benzimidazole (Wako) (approximately 2000 animals/9-cm plate) and cultured at 20°C for 6 to 7 days. Because *tm246(vps-45) *mutants exhibit a temperature-sensitive phenotype, only *tm246 *mutants carrying *vps-45 *rescue transgenes can survive at 20°C (positive selection). In contrast, because *ben-1(tm234) *mutants are resistant to benzimidazole, *tm234 *mutants carrying *ben-1 *transgenes are sensitive and unable to survive on benzimidazole-containing plates (negative selection). Transformed animals were cloned and further cultured on benzimidazole-containing plates over several generations. To ensure that each strain was established independently, only one transformant was picked up from each selection plate. Transformants were selected by PCR#A and PCR#B (as shown in Figure 1), which amplify the 5' and 3' regions of HBG-LoxP-vps-45-LoxP, respectively. Primers used are listed in Additional file 4, Table S2.

### Genomic-excision using Cre recombinase mRNA

Cre recombinase cDNA was amplified from AxCANCre (TaKaRa) [20] and cloned into the pGEMT vector. Cre recombinase RNA was synthesized *in vitro *using a mMESSAGE mMACHINE T7 kit (Ambion). Synthesized RNA was purified by phenol/chloroform extraction followed by isoamyl alcohol precipitation. Poly-A tailing was performed using Yeast Poly(A) Polymerase (74225Y, affymetrix). Poly-A tailed RNA was purified by phenol/chloroform extraction followed by ethanol precipitation. Cre recombinase mRNA was injected at 1 μg/ml along with *P*_*myo-2*_*::venus *as an injection marker (67 ng/ml) into integrant animals carrying a floxed *vps-45 *mini gene. F1 animals were genotyped by PCR#1 to detect Cre-mediated excision. F2 self-progenies from heterozygotes for the Cre-excised allele were genotyped to obtain homozygotes for the Cre-excised allele. Homozygous lines were further tested by PCR#2 to detect non-excised allele (as shown in Figure 2). Primers used are listed in Additional file 4, Table S2.

### Quantitative PCR

Genome DNA was isolated from adult animals using DNeasy Tissue & Blood kit (QIAGEN). Quantitative PCR was performed in a 7500 Real-time Thermal cycler (Applied Biosystems) using the Power SYBR master mix (Applied Biosystems) with the following parameters: 95°C for 10 min and 40 cycles of 95°C for 5 s, 55°C for 10 s and 72°C for 34 s. All data were normalized to the *act-2 *gene. Primers were designed within an exon for each gene using the Primer3 software. The primers used are listed in Additional file 4, Table S2.

### SNP mapping

To map insertion sites, SNPs between the Hawaiian strain CB4856 [21] and the parent strain Bristol N2, were used. Worm lysis and SNP mapping were based on the procedures described previously [22] with some modification. Briefly, N2-backround strains of interest were outcrossed 8 to 12 times with CB4856 and assayed for linkage between the HBG-loxP sequence (detected by PCR#1) and SNPs.

### Microscopy

Differential interference contrast and fluorescence images were obtained using a BX51 microscope equipped with a DP30BW CCD camera (Olympus Optical Co., Ltd).

## Authors' contributions

EKN, HK and SM conceived and designed the UV/TMP integration method, carried out and supervised the experiments, and drafted the manuscript. OF carried out the molecular genetic studies. MO, KGA, SY and SH participated in the design of study and the construction of selection markers. All authors read and approved the final manuscript.

## Supplementary Material

Additional file 1 **Figure S1**. A positive-negative selection scheme. The *tm246(vps-45) *mutants exhibited a temperature-sensitive phenotype. Only *tm246 *mutants carrying *vps-45 *rescue transgenes survived at 20°C (positive selection). The *tm234(ben-1) *mutants were resistant to benzimidazole. *tm234 *mutants carrying *ben-1 *rescue transgenes are sensitive and unable to survive on benzimidazole-containing plates (negative selection). Phenotypes of *tm234;tm246 *were rescued by transgenes, enabling the Ex line to survive at 20°C. The parent Ex animals were treated by UV/TMP, resulting in multi-copy insertion (Is), low-copy Is, and Ex arrays. Because multi-copy Is animals and Ex animals were highly likely to have the *ben-1 *transgene, only low-copy Is animals carrying the *vps-45 *and not the *ben-1 *transgene were selected. Click here for file

Additional file 2 **Table S1**. Comparison of single- or low-copy integration methods. Click here for file

Additional file 3 **Figure S2**. Heat-shock induction of *Venus *mRNA in integrant strains. Relative expression of *venus *mRNA determined by quantitative RT-PCR. Total RNA was extracted from a 100-μl pellet of worms collected from 0-min, 30-min, and 1-h culture at 20°C after heat-shock at 32°C for 1 h. The expression of mRNA (normalized to *act-2*) is presented as a ratio to heat-shock (-) control. *tmIs893 *and *tmIs894 *were examined. Click here for file

Additional file 4 **Table S2**. PCR primers. Click here for file
