# Supplementary figures and images for: Single/low-copy integration of transgenes in Caenorhabditis elegans using an ultraviolet trimethylpsoralen method
Source: BMC Biotechnol. 2012 Jan 5;12:1. doi: 10.1186/1472-6750-12-1 (PMC3262153; doi:10.1186/1472-6750-12-1)

# Positive-Negative Selection

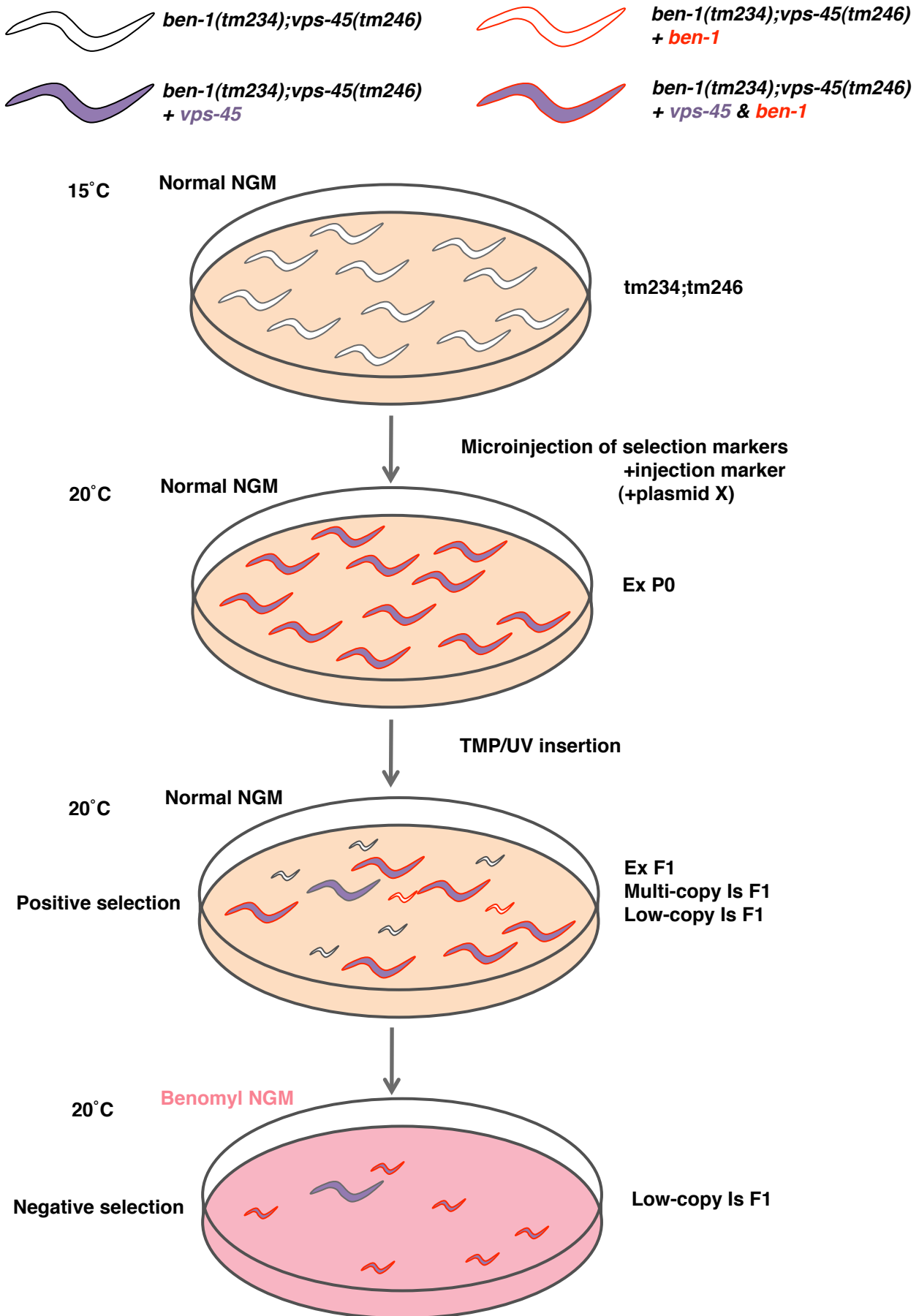

Supplementary Figure1

Supplement: Additional file 1 — Figure S1. A positive-negative selection scheme. The tm246(vps-45) mutants exhibited a temperature-sensitive phenotype. Only tm246 mutants carrying vps-45 rescue transgenes survived at 20°C (positive selection). The tm234(ben-1) mutants were resistant to benzimidazole. tm234 mutants carrying ben-1 rescue transgenes are sensitive and unable to survive on benzimidazole-containing plates (negative selection). Phenotypes of tm234;tm246 were rescued by transgenes, enabling the Ex line to survive at 20°C. The parent Ex animals were treated by UV/TMP, resulting in multi-copy insertion (Is), low-copy Is, and Ex arrays. Because multi-copy Is animals and Ex animals were highly likely to have the ben-1 transgene, only low-copy Is animals carrying the vps-45 and not the ben-1 transgene were selected. [file 1472-6750-12-1-S1.PDF]

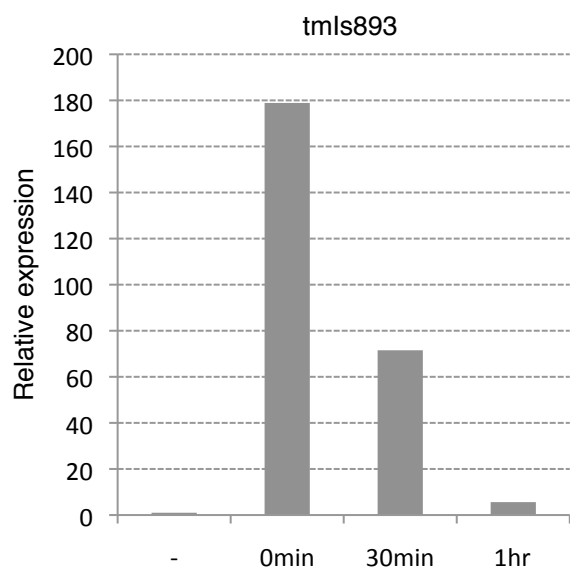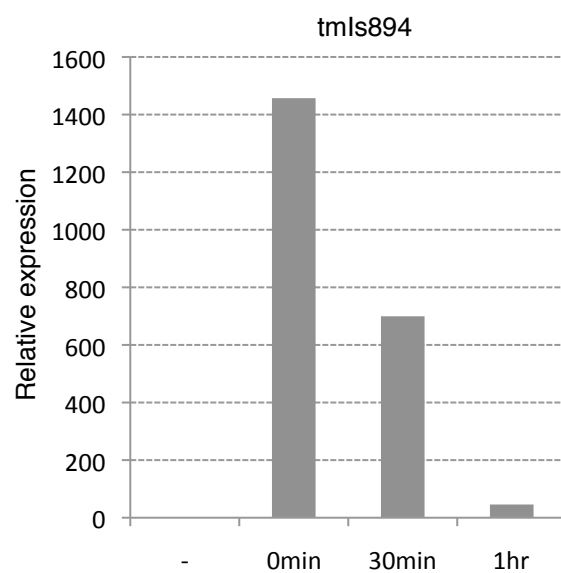

Supplementary Figure 2

Supplement: Additional file 3 — Figure S2. Heat-shock induction of Venus mRNA in integrant strains. Relative expression of venus mRNA determined by quantitative RT-PCR. Total RNA was extracted from a 100-μl pellet of worms collected from 0-min, 30-min, and 1-h culture at 20°C after heat-shock at 32°C for 1 h. The expression of mRNA (normalized to act-2) is presented as a ratio to heat-shock (-) control. tmIs893 and tmIs894 were examined. [file 1472-6750-12-1-S3.PDF]
