# Supplementary material for: Single/low-copy integration of transgenes in Caenorhabditis elegans using an ultraviolet trimethylpsoralen method
Source: BMC Biotechnol. 2012 Jan 5;12:1. doi: 10.1186/1472-6750-12-1 (PMC3262153; doi:10.1186/1472-6750-12-1)
Supplement: Additional file 2 — Table S1. Comparison of single- or low-copy integration methods. [file 1472-6750-12-1-S2.DOC]

**Table S1** Comparison of single- or low-copy integration methods

| Method | Insertion | Recipient background | Positive selection | Negative selection | Frequency | Ref. |
| --- | --- | --- | --- | --- | --- | --- |
|  | (Copy number) |  | (Phenotype rescued) | (Phenotype conferred) | (Integration F1/P0 animals) |  |
| Bombardment | Random | *unc-119(ed3)III* | *unc-119* | high-copy *sup-7* or none | 2 / ~220,000 (0.0009%) | 6 |
|  | (Low) |  | (Unc, Daf-d) | (Toxic) | ~ 7 / ~200,000 (0.0035%) |  |
|  |  |  |  |  |  |  |
| MosSCI | Site-directed | *Mos1 alleles* | *unc-119* | *twk-18(gf)* or none | 5 / 61 (8%) ~ 13 / 63 (21%) | 7 |
|  | (Single) | *;unc-119(ed3)III* | (Unc, Daf-d) | (Paralyzed @25˚C) |  |  |
|  |  |  |  |  |  |  |
| UV/TMP | Random | *ben-1(tm234)III* | *vps-45* | *ben-1* rescue | 4 / 16,000 (0.025%) | This |
|  | (Single, low) | *;vps-45(tm246)X* | (Gro, Let @>20˚C) | (Benzimidazole-sensitive) | ~ 22 / 10,400 (0.21%) | study |
