# Supplementary material for: Single/low-copy integration of transgenes in Caenorhabditis elegans using an ultraviolet trimethylpsoralen method
Source: BMC Biotechnol. 2012 Jan 5;12:1. doi: 10.1186/1472-6750-12-1 (PMC3262153; doi:10.1186/1472-6750-12-1)
Supplement: Additional file 4 — Table S2. PCR primers. [file 1472-6750-12-1-S4.DOC]

**Table S2** PCR primers

| Primer | Sequence |
| --- | --- |
| PCR#A forward | CGCCAGGGTTTTCCCAGTCACGAC |
| PCR#A reverse | CTTGACTGCACGAGATCCAT |
| PCR#B forward | TCAGTGGCATTCTACAGATG |
| PCR#B reverse | TGACCATGATTACGCCAAGC |
| PCR#C forward | TAGAGAATGAACAGTAAGCACT |
| PCR#C reverse | TTACTTGTACAGCTCGTCCA |
| PCR#1 forward | GGAGGACAAGGCTACTATCA |
| PCR#1 reverse | AGAGCGCCCAATACGCAAAC |
| PCR#2 forward | GGAGGACAAGGCTACTATCA |
| PCR#2 reverse | CTCCTGAAGCTCTTTCACCA |
| act-2_qPCR forward | ATCGTCCTCGACTCTGGAGAT |
| act-2_qPCR reverse | TCACGTCCAGCCAAGTCAAG |
| vps-45_qPCR1 forward | AACAGTGTCATGCGCATTTG |
| vps-45_qPCR1 reverse | CTCCTGGAGCTCTTTCACCA |
| vps-45_qPCR2 forward | TGCGTGAGGTTCAAGAAGTG |
| vps-45_qPCR2 reverse | AACAGCTGGAGCCTTTTTCA |
| hsp-16.1p_qPCR3 forward | AGGTGCAAAGAGACGCAGAT |
| hsp-16.1p_qPCR3 reverse | TGTTTGGTTCGGTTTTGTCA |
